# Low-density scalp electrical source imaging of the ictal onset zone network using source coherence maps

**DOI:** 10.3389/fneur.2024.1483977

**Published:** 2024-12-19

**Authors:** Parnia Sadeghzadeh, Alexander Freibauer, Rajesh RamachandranNair, Robyn Whitney, Mutaz Al Nassar, Puneet Jain, Elizabeth Donner, Ayako Ochi, Kevin C. Jones

**Affiliations:** ^1^Division of Neurology, Department of Pediatrics, McMaster Children’s Hospital, Hamilton, ON, Canada; ^2^Division of Neurology, Department of Pediatrics, BC Children’s Hospital, Vancouver, BC, Canada; ^3^Division of Neuroimaging, Department of Diagnostic Imaging, McMaster Children’s Hospital, Hamilton, ON, Canada; ^4^Division of Neurology, Department of Pediatrics, The Hospital for Sick Children, Toronto, ON, Canada

**Keywords:** electrical source imaging, ictal onset zone, source coherence maps, low density, epilepsy surgery

## Abstract

**Introduction:**

This study investigated low-density scalp electrical source imaging of the ictal onset zone and interictal spike ripple high-frequency oscillation networks using source coherence maps in the pediatric epilepsy surgical workup. Intracranial monitoring, the gold standard for determining epileptogenic zones, has limited spatial sampling. Source coherence analysis presents a promising new non-invasive technique.

**Methods:**

This was a retrospective review of 12 patients who underwent focal resections. Source coherence maps were generated using standardized low-resolution electromagnetic tomography and concordance to resection margins was assessed, noting outcomes at 3 years post-surgery.

**Results:**

Ictal source coherence maps were performed in 7/12 patients. Six of seven included the surgical resection. Five of seven cases were seizure free post-resection. Interictal spike ripple electrical source imaging and interictal spike ripple high-frequency oscillation networks using source coherence maps were performed for three cases, with two of three included in the resection and all three were seizure free.

**Discussion:**

These findings may provide proof of principle supporting low-density scalp electrical source imaging of the ictal onset zone and spike ripple network using source coherence maps. This promising method is complementary to ictal and interictal electrical source imaging in the pediatric epilepsy surgical workup, guiding electrode placement for intracranial monitoring to identify the epileptogenic zone.

## Introduction

People living with epilepsy often respond to treatment with antiseizure medications; however, approximately 20% will develop drug-resistant epilepsy (1). Epilepsy surgery is the treatment of choice in patients with drug-resistant epilepsy (2, 3). Selected candidates for epilepsy surgery must undergo a presurgical workup, to identifying the epileptogenic zone (EZ), the area of the brain which if successfully resected results in seizure freedom (4). The EZ is inferred from the localization of various zones by diagnostic techniques and verified with post-surgical seizure freedom. The symptomatogenic zone, which is the area responsible for clinical symptoms, is determined by ictal semiology. EEG is used to determine the ictal onset zone, which is the area of the brain that generates the EEG signal at the onset of seizures. EEG spikes or magnetoencephalography (MEG) determine the irritative zone, which is usually broader than the epileptogenic zone. An epileptogenic lesion, which is often included in the resection margin, is determined by magnetic resonance imaging (MRI). F-fluorodeoxyglucose positron emission tomography (FDG-PET), scan is utilized to determine the functional deficit zone, which includes areas of hypometabolism. Finally, the eloquent cortex is localized with functional magnetic resonance imaging (fMRI) or the Wada test to minimize deficits post-operatively. These diagnostic tests are then brought together in the localization hypothesis, with the ultimate goal of deciding whether a patient is an appropriate resective surgical candidate or not. Standard protocol indicates obtaining a history, video EEG, 3D volumetric MRI and neuropsychology assessment. In more complex cases, an FDG PET, Single-photon emission-computerized tomography (SPECT), Electrical source imaging ESI, fMRI, Wada test or MEG may be indicated (5). The utilization of these various non-invasive diagnostic techniques in epilepsy surgery workup can be quite costly, resource intensive, and inaccessible. Intracranial monitoring remains the gold standard in determining the precise location and boundary of the EZ for resection. However, intracranial monitoring is invasive and limited in its spatial sampling, with electrode placement being crucial to avoid sampling error (5, 6).

Epilepsy surgery outcome (International League Against Epilepsy ILAE Class 1–2 or Engel Class 1) in children is influenced by the type of lesion resected, the location of surgery, the extent of the surgery, and the involvement of the eloquent cortex. Histopathology is an important prognostic factor for the outcome of epilepsy surgery. At 2-year follow-up, the best seizure outcomes were 77.5% for low-grade epilepsy-associated neuroepithelial tumors (LEAT), 74% for vascular malformations, and 71.5% for hippocampal sclerosis. The worst seizure outcomes at 2 years were 50% for focal cortical dysplasia (FCD) type I or mild malformation of cortical development (MCD) and 53% for MCD other (7). Temporal or extratemporal seizure location in combination with etiology (LEAT vs. FCD) is also an important predictor of seizure outcome. For LEAT, seizure freedom was high, regardless of temporal or extratemporal location; however, for FCD, temporal lobe location had a higher proportion of seizure freedom than extratemporal locations. Complete resection of the EZ was also more likely to result in seizure freedom. This finding is more pronounced for extratemporal lobe epilepsy (8–12). FCD can have worse outcomes than tumors as they can appear non-lesional on MRI or have multilobar epileptogenic zones. Additional preoperative diagnostic testing to identify the EZ may be associated with better seizure outcomes in non-lesional MRI cases and extra temporal lobe epilepsy (13, 14).

Finally, delineating eloquent cortex predicts outcome as the involvement of the eloquent cortex within the EZ may limit the extent of the surgical resection of the EZ resulting in suboptimal seizure outcomes. There is a clinical need within epilepsy surgery workup for more accessible, non-invasive diagnostic techniques that effectively localize the EZ.

ESI presents a promising method for the localization of the EZ. ESI involves the utilization of computational analysis of EEG recordings to generate current density reconstruction maps (15). There is a growing body of literature supporting the accuracy of ictal and interictal ESI in source localization for epilepsy surgery workup (16–23). Ictal ESI has been supported as a promising localization technique in various populations, including patients with focal epilepsy and TLE (24–27). In regard to interictal ESI, Brodbeck et al. published a landmark paper in 2011 demonstrating the validity and utility of interictal ESI in a large prospective study for epilepsy surgery workup (28). ESI has also been demonstrated to yield non-redundant information. Data obtained from low-density (LD) ictal and interictal ESI and high-density (HD) interictal ESI have been shown to change presurgical management plans for over 20% of patients (29). HD EEG is more practical for localizing interictal discharges with ESI during short studies. At the same time, LD EEG is preferable for the prolonged video EEG recordings required to capture seizures and determine the ictal onset zone with ESI in children. In addition, literature demonstrates extensive support for ESI with various methodologies, populations, and contexts, with ESI being shown to have greater concordance in frontal lobe epilepsy, MRI-negative epilepsy, and patients with malformation cortical development (30–49).

Research into ESI has primarily focused on estimating the ictal onset using a source of maximum power. However, epilepsy is a network disease, meaning it is imperative to consider the role that connectivity between brain regions plays at seizure onset (50). Source coherence maps address this by providing additional functional connectivity analysis to ESI. Source coherence maps utilize EEG analysis to determine the functional connectivity via temporal correlation of neuronal activity between different brain regions to estimate the seizure onset zone (15). Ding et al. were the first to combine ESI with connectivity analysis to estimate the ictal onset zone, and there is a growing body of evidence supporting the utilization of functional connectivity with ESI to effectively localize the ictal onset zone (51–57). HD-EEG with connectivity analysis estimated the seizure onset zone within resections with high accuracy and demonstrated its superiority to ESI alone (54, 56–58). LD-EEG has been used to create functional networks, which predict the hemisphere of surgery (59). There has also been support in literature for the utilization of ESI to estimate the functional connectivity of epileptogenic networks through implantation of virtual sensors. This presents a method to potentially replicate the ability of intracranial EEG to map the epileptogenic network (60–63).

Pathological high-frequency oscillations (HFOs), also present as a promising biomarker of epileptogenic tissue (64, 65). Analyzing interictal EEGs during sleep for HFOs that co-occur with spikes can lead to accurate identification of pathological HFOs (64, 66). A recent study by Mooij et al. demonstrated that ripples could be classified as physiological or pathological with 98% accuracy using scalp EEG (67).

Specifically, the identification of HFOs using scalp EEGs is of great interest clinically, given the accessibility of this method for epilepsy surgery workup (66, 68–73). Another novel consideration is using source imaging or localization to analyze HFOs. ESI has thus far only been used by seven studies to date to analyze HFOs, all of which utilize HD-EEG and largely focus on a patient population with drug-refractory epilepsy and surgical resection. HFOs as well as interictal ripples have been localized using source imaging with high accuracy (66, 70–72).

To date, studies have not yet examined functional connectivity networks in conjunction with ESI to analyze HFOs for epilepsy surgery evaluation in children. Given the novelty of HFOs, there is a clear need for further research within this area, such as utilizing LD-EEG and specifically looking at source imaging and functional connectivity networks.

This study builds on Thurairajah et al. in which promising concordance of the ictal LD-ESI localizations to the EZ was demonstrated by comparing the ictal LD-ESI to the focal resection margins on neuroimaging and noting postoperative outcomes (22). The objective was to investigate epileptic network localization through source coherence maps using LD-ESI of the ictal onset zone and interictal spike ripple high-frequency oscillation networks in children undergoing epilepsy surgical workup.

## Methods

This was a retrospective study of consecutive children who completed a pre-surgical evaluation between July 2014 and July 2019 within the Comprehensive Pediatric Epilepsy Programs at McMaster Children’s Hospital (Hamilton, Ontario) and the Hospital for Sick Children (Toronto, Ontario) and then underwent focal epilepsy surgery. The inclusion criteria and patient data, including EEG, MRI, FDG PET, and MEG acquisition and the initial interictal and ictal EEG source imaging analysis, have previously been described in Thurairajah et al. (22).

In addition, EEG source coherence analysis with Curry 9 software by Compumedics Neuroscan was performed using scalp EEG data and individual volumetric T1 MRI images. Noise estimation was adjusted automatically or with a user-defined noise level. Source coherence in Curry is intended for use with fixed dipoles and current density reconstructions (sLORETA) using a 3D grid model with grid spacing set to 15 mm to reduce the large number of coherence links. Source coherence prefers less than a few thousand source locations for numerical reasons. The results are presented as current density reconstruction currents clipped below a user-defined percentage to indicate the degree of coherence between 0 and 1. Sources with strengths below the clipping threshold are not included in the coherence analysis. The clipping threshold determines the percentage of maximum source strength analyzed. Minimum and maximum lag times are set to define the range of lag times in milliseconds included in the analysis. Zero lag removal is switched on to avoid spurious results due to cortical volume conduction.

### Ictal onset zone source coherence

The initial 1–3 s of the ictal onset zone and the frequency band of interest (delta 0.5–3 Hz, theta 4- < 8 Hz, alpha 8–12 Hz, and beta 12–30 Hz) for a representative seizure from each case was identified by epileptologist (KJ) by review of the EEG in standard bipolar and referential montages. Ictal onset zone source coherence maps were generated using a boundary element head model using a source location 3d grid with grid spacing of 15 mm, and a fixed standardized low resolution electromagnetic tomography (sLORETA) current density map with fixed dipoles, and zero lag removal switched on (to exclude volume conduction) with a source minimum lag time range of 5 ms and maximum lag time range of 100 ms and a minimum distance of 5 mm clipped below 70%. Source coherence connectivity maps and lag times were depicted on a three-dimensional head model and reviewed by an epileptologist (KJ) for the presence or absence of source connectivity networks.

### Interictal source coherence

Representative focal interictal discharges for each case were marked by an epileptologist (KJ) by review of EEG in standard bipolar and referential montages. A 1–30-Hz filter was applied. The onset to the peak of the averaged spikes for each case was analyzed. Interictal spike source coherence maps were generated using the same method as per ictal onset source coherence maps. Interictal ripple ESI was performed on EEG recorded at 1024 Hz, by applying an 80–200-Hz filter over the same averaged focal interictal discharges for each case. A 200 ms epoch at the averaged spikes was delineated and a short time fast Fourier transform (STFFT) analysis was applied to ensure ripple activity during the marked epoch. Independent component analysis was performed using a current density model sLORETA clipped below 80%. Interictal ripple source coherence was performed by applying an 80–200 Hz on the same averaged focal interictal discharges for each case. Source coherence maps were generated and reviewed as per the ictal onset zone source coherence maps.

## Outcomes

Postoperative outcomes were assessed according to the International League Against Epilepsy (ILAE) surgical outcomes, which looks at outcomes after a defined period, in this case at the 3-year follow-up. Seizure outcomes were deemed to be favorable (ILAE Class 1–2) if there was complete seizure freedom or only auras and no other seizures after surgery. This corresponds to Class I of the Engel classification (free of disabling seizures). Seizure outcome was considered unfavorable (ILAE Class 3–6) if there were 1 to 3 seizure days per year with or without auras or more than 3 seizure days per year, which corresponds to Engel Class II-IV if there were rare disabling seizures or worthwhile improvement or no worthwhile improvement (74).

## Reference standard and definition of EZ

The EZ was defined as the resected region in seizure-free patients after 3 years. Concordance with the EZ was established if the irritative zone or ictal onset zone was localized within the surgical resection margins of patients with favorable seizure outcomes (ILAE Class 1–2). Ictal and interictal source connectivity and interictal HFO source connectivity network concordance were established if the epileptic network overlapped the resection margins of patients with favorable seizure outcomes (ILAE Class 1–2). The clinical information was available to the index test performer (KJ), and clinical information and index test results were available to the reference standard assessor (KJ).

## Results

### Focal resections

**Figure 1:**
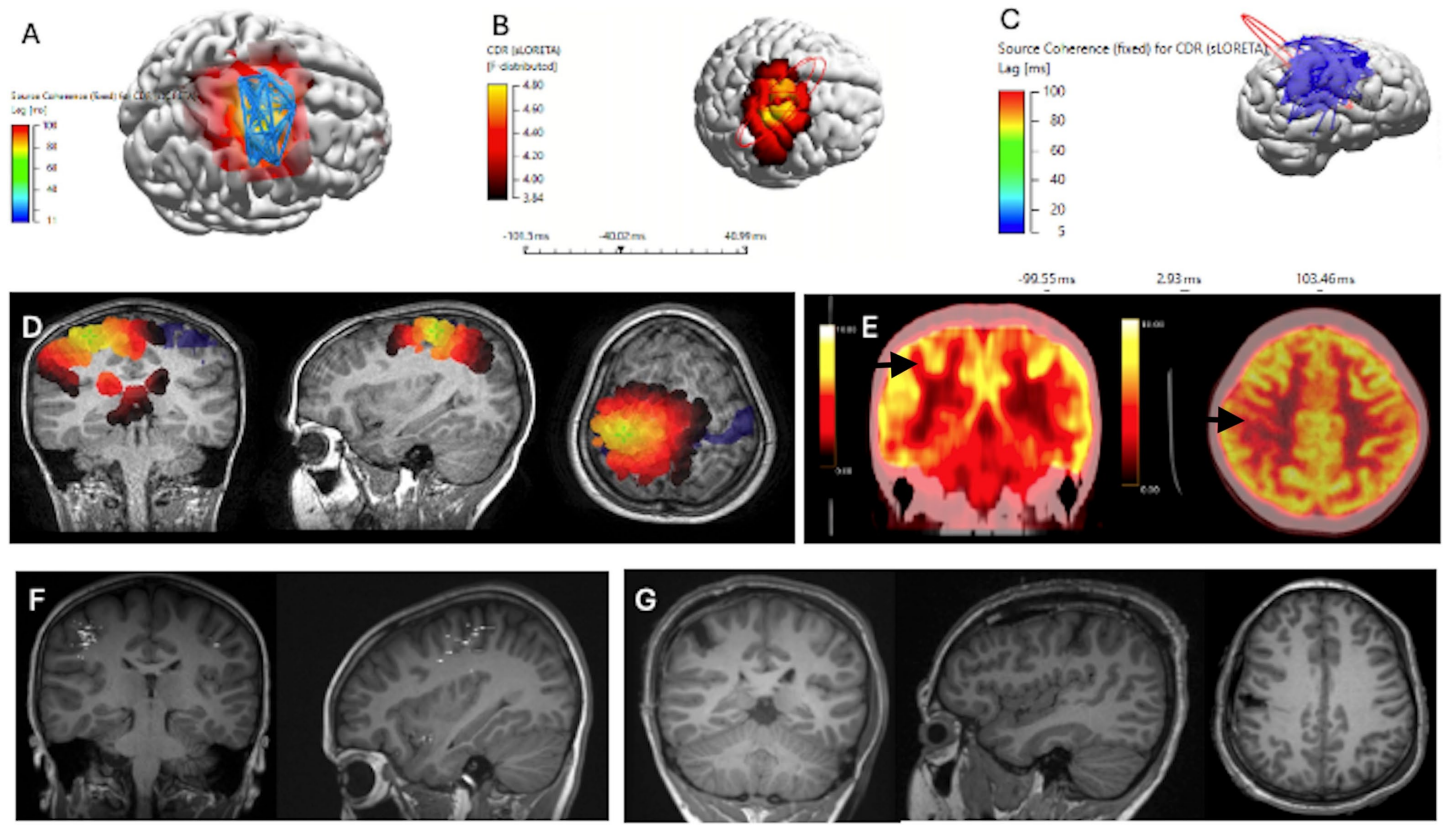
Concordant Localization of Ictal ESI Source Coherence, Interictal HFO ESI and Interictal HFO Source Coherence to Resection Margins, for ILAE Class 1 Patient, alongside MEG and FDG PET scans (Case 2). Patient underwent a lesionectomy of a right post-central gyrus lesion. **(A)** Ictal ESI Source Coherence Map localizing to the right pre-central and post-central gyrus. **(B)** Interictal HFO ESI localizing to the right post-central gyrus. **(C)** Interictal HFO Source Coherence map identifying a network localizing to the right parietal, central and temporal regions. **(D)** sLORETA source localization through ICA, clipped at 75%. **(E)** FDG-PET scans identifying a region of hypometabolism within the right post-central gyrus. **(F)** Interictal MEG Dipole Cluster localizing to the right frontal, temporal, and parietal regions. **(G)** T1 MRI Images of Post-Operative Resection Margins with Coronal, Sagittal and Axial views.

**Figure 2:**
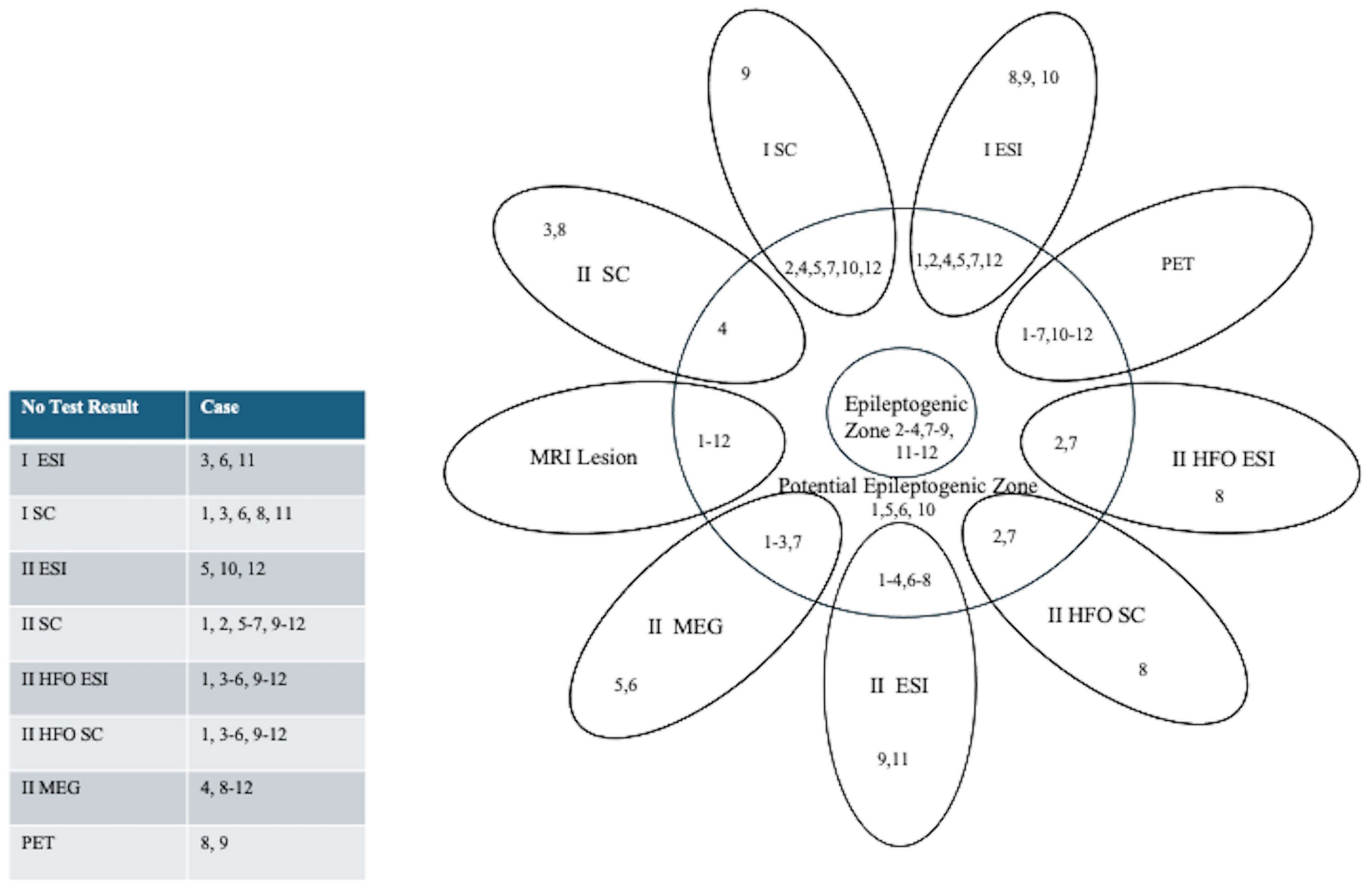
Summary Diagram: Concordance of Pre-Surgical Work-Up, ESI and Source Coherence Localization to the Potential Epileptogenic Zone (resection). Patients are indicated by their corresponding case number. The epileptogenic zone was defined as the resected region in seizure-free patients after three years. Numbers overlapping with the potential epileptogenic zone (resection) indicate concordant imaging results, which were established if the irritative zone or ictal onset zone was localized within the surgical resection margins. Those outside the potential epileptogenic zone indicate discordant results. A table on the left indicates a list of cases without test results for each diagnostic modality.

Twelve children (seven female, median age 11 years, interquartile range 8.5 years) underwent focal resections. Age, gender, MRI lesion, FDG-PET hypometabolism, MEG dipole clusters, ictal ESI localization, ictal source coherence, interictal ESI localization, interictal source coherence, interictal HFO ESI, interictal HFO source coherence, intracranial monitoring, surgical operation, pathology, and postoperative outcomes are outlined in Table 1. An illustrative case is depicted in Figure 1. A summary of the concordance of the presurgical workup, ESI and source coherence localizations with the potential EZ (resection) of each case (indicated by their case numbers) is depicted by the Venn diagram in Figure 2. The potential EZ (resection) typically fully included the MRI lesions (unless the eloquent cortex was involved) and partially included the other diagnostic zones. Diagnostic zones that were not at all within the potential EZ (resection) were also depicted on the Venn diagram in Figure 2. A list of cases without test results for each diagnostic modality is also included in Figure 2.

**Table 1:** Clinical profiles.

Case (*n* = 12)	Age (Median 11; IQR 8.5)	Sex (7 female)	MRI	PET	MEG	I ESI	I SC	II ESI	II SC	II HFO ESI	II HFO SC	I ESI resection zone concordance	Concordant data	Discordant data	Intracranial monitoring	Surgical operation	Pathology	ILAE seizure outcome class at 3 years
1	17	M	R temporal	R temporal	R temporal	R temporal	NA	R temporal	NA	NA	NA	Yes	MRI, PET, MEG, I ESI, II ESI	No	Subdural grid	R temporal lobectomy	Heterotopic gray matter	3
2	12	F	R post central gyrus	R post central gyrus	R frontal temporal parietal	R postcentral gyrus	R post central -precentral gyrus	R postcentral gyrus	NA	R postcentral gyrus	R parietal central temporal network	Yes	MRI, PET, MEG, I ESI, I SC ESI, II ESI, II HFO ESI, II HFO SC ESI	No	Subdural grid/ depth electrodes	Right postcentral gyrus lesionectomy	FCD II B	1
3	1	F	R hemisphere hypoplastic	R temporal occipital	R posterior quadrant	NA	NA	R temporal	Generalized	NA	NA	NA	MRI, PET, MEG, II ESI	II SC ESI	No	R posterior quadrant disconnection R temporal lobectomy	Unknown	1
4	16	F	R occipital	R occipital temporal parietal	NA	R parietal	R occipital parietal	R occipital	Right > left parietal occipital	NA	NA	Yes	MRI, PET, I ESI, I SC ESI, II ESI, II SC ESI	No	Subdural grid, strips, depth electrodes	R occipital, parietal lesionectomy	Ganglioglioma	1
5	4	M	L cingulate gyrus	L cingulate gyrus	R peri-sylvian	L caudate	L central temporal parietal	NA	NA	NA	NA	Yes	MRI, PET, I ESI, I SC ESI	MEG	Depth electrodes	L cingulate lesionectomy	Ganglioglioma	4
6	17	F	R temporal orbital frontal	R temporal	R rolandic insular	NA	NA	R temporal	NA	NA	NA	NA	MRI, PET, II ESI	MEG,	Stereotactic EEG	R anterior temporal lobectomy, R orbital frontal resection	Subpial gliosis, oligodendrogliosis	4
7	13	F	L parietal, post central gyrus	L post central gyrus	L rolandic	L parietal	L parietal	L parietal	NA	L parietal	L Parietal occipital > right	Yes	MRI, PET, MEG, I ESI, I SC ESI, II ESI, II HFO ESI, II HFO SC ESI	No	Subdural grid/ depth electrodes	L post central gyrus lesionectomy	FCD II A	1
8	12	M	L orbital frontal	NA	NA	L frontal	NA	L frontal	Generalized	L temporal	L frontal	No	MRI, II ESI,	I ESI, II SC ESI, II HFO ESI, II HFO SC ESI	No	L gyrus rectus lesionectomy	FCD IIIB	1
9	10	F	R cingulate gyrus	NA	NA	R temporal, insular	R hemisphere	R temporal	NA	NA	NA	No	MRI	I ESI, I SC ESI, II ESI	No	R cingulate gyrus lesionectomy	DNET	1
10	8	M	R parietal	R parietal occipital	NA	R temporal	R occipital parietal temporal	NA	NA	NA	NA	No	MRI, PET, I SC ESI	I ESI	Stereotactic EEG	R parietal lesionectomy	FCD IIB	4
11	3	F	L temporal, insular	L temporal	NA	NA	NA	L frontal temporal parietal	NA	NA	NA	NA	MRI, PET	II ESI	No	L temporal lobectomy	Ganglioglioma	1
12	8	M	R mesial temporal	R temporal	NA	R posterior temporal	R occipital parietal temporal	NA	NA	NA	NA	Yes	MRI, PET, I ESI, I SC ESI	No	No	R temporal lobectomy	Gliosis, hippocampal sclerosis	1

NA, not applicable; M, male; F, female; R, right; L, left; I MRI, magnetic resonance imaging; PET, positron emission tomography; MEG, magnetoencephalography; I ESI, ictal electrical source imaging; I SC, ictal source coherence; II ESI, interictal electrical source imaging; II HFO ESI, interictal high frequency oscillation (Ripples 80–200 Hz) electrical source imaging; II HFO SC, interictal high frequency oscillation (Ripples 80–200 Hz) source coherence; FCD, focal cortical dysplasia; DNET, dysembryoplastic neuroepithelial tumour.

All cases had MRIs available for review, ten cases had FDG-PET scans (cases 8 and 9 excluded), and six had MEG scans available for review (cases 4, 8, 9, 10, 11, and 12 excluded). Of the twelve cases with focal resections, ten were seizure-free 1 year following the surgery or ILAE Class 1 (cases 2, 3, 4, 6, 7, 8, 9, 10, 11, and 12 included). Two cases (cases 1 and 5) were not seizure free at 1 year. These were categorized as ILAE Class 3 and Class 4, respectively. After 3 years, eight cases remained seizure free (cases 2, 3, 4, 7, 8, 9, 11, and 12 included). Two cases (cases 6 and 10) relapsed after 1 year, both were categorized as ILAE Class 4.

### Ictal ESI

The ictal onset zone was localized through ESI for nine of the twelve cases (cases 1, 2, 4, 5, 7–10, and 12) with cases 3, 6, and 11 having inconclusive localization. In case 3, this was due to the presence of epileptic spasms, which made it challenging to visualize the ictal onset zone with LD ESI due to the brief duration of the seizure and the ictal morphology. In case 6, the presence of significant muscle artifacts limited the utility of ictal ESI. In case 11, the ictal source was located deep within the basal ganglia. Ictal ESI was concordant with ictal source coherence (cases 2, 4, 5, 7, and 12 included) where data were available (cases 2, 4, 5, 7, 9, 10, and 12). Ictal ESI was concordant with interictal source coherence (case 4) where data were available (cases 3, 4, and 8). Cases with ictal ESI, interictal HFO ESI, and interictal HFO source coherence showed concordance in two cases (cases 2 and 7 included), but not in one (case 8).

### Interictal ESI

The irritative zone was localized for nine of twelve cases using interictal ESI (cases 1, 2, 3, 4, 6, 7, 8, 9, and 11). Cases 5, 10, and 12 have no test results as there were no spikes detected on scalp EEG. Interictal ESI was concordant with ictal source coherence (cases 2, 4, and 7 included) from the available cases (2, 4, 5, 7, 9, 10, 12, and) and interictal source coherence (cases 4) where data were available (cases 3, 4, and 8). Cases with interictal ESI, interictal HFO ESI, and interictal HFO source coherence ESI showed concordance in two cases (cases 2 and 7 included), but not in one case (case 8).

### Ictal source coherence

Ictal source coherence maps were performed in seven of twelve patients (cases 2, 4, 5, 7, 9, 10, and 12 included). Six of seven source coherence maps included the surgical resection. Five of seven cases were seizure free post-resection excluding case 5 (Class 4 outcome at 1 year) and case 10 (Class 4 outcome at 3 years). Cases were concordant with MRI, PET, ictal ESI (except case 10), interictal ESI, interictal source coherence, interictal HFO ESI, and interictal HFO source coherence where available. All cases had ictal source coherence maps concordant with MEG, except case 5, in which MEG was discordant with all other data and the surgical resection. There were no test results for ictal source coherence (cases 1, 3, 6, 8, and 11) as cases 1 and 8 did not demonstrate source coherence networks >70% and cases 3, 6, and 11 had inconclusive ictal EEG localization.

### Interictal source coherence

Interictal source coherence maps were created in three of twelve patients (cases 3, 4, and 8 included). Cases 3 and 8 displayed generalized activity. Case 4 demonstrated a network involving the parietal occipital region, more evident over the right hemisphere. This included the surgical resection site, and the patient was seizure-free postop. The interictal source coherence map for case 4 was also concordant with available MRI, PET, ictal ESI, and interictal ESI. Source coherence mapping is a postprocessing tool for source analysis results. There were no test results for cases 1, 2, 5–7, and 9–12 as cases 1, 2, 6, 7, 9, and 11 did not demonstrate spike source coherence networks >70% (which was the threshold for inclusion) and cases 5, 10, and 12 who had no spikes detected on scalp EEG.

### Interictal HFO ESI

This was performed in three of twelve patients (cases 2, 7, and 8, included). Interictal HFO ESI included the surgical resection for cases 2 and 7, which were all seizure free postresection. Interictal HFO ESI was concordant with MRI, interictal ESI, and interictal HFO source coherence for two of three cases, as well as PET and MEG, which were available for cases 2 and 7. Cases 2 and 7 were also concordant to ictal ESI; however, case 8 was not. Cases 1, 3–6, and 9–12 had no test result. Ripple frequencies were not recorded for cases 3 and 4. Interictal spikes were not detected for cases 5, 10, and 12. In addition, no distinct HFO band was seen on STFFT for cases 1, 6, 9, and 11.

### Interictal HFO source coherence

These maps were generated for three of twelve patients, who had interictal HFO ESI with distinct HFO bands seen on SFFT (2, 7, and 8 included). Cases 2 and 7 included the surgical resection and were seizure free postresection. Interictal HFO source coherence was concordant with MRI, interictal ESI, ictal ESI, and interictal HFO ESI for two of three cases, as well as FDG-PET and MEG which were available for cases 2 and 7. Cases 1, 3–6, and 9–12 had no test results for the same reasons that interictal HFO ESI did not.

### Interictal magnetic source imaging with MEG

These scans were conducted in nine of twelve cases (cases 1, 2, 3, 4, 5, 6, 7, 10, and 11). Interictal magnetic source imaging dipole clusters were localized for six of nine cases (cases 1, 2, 3, 5, 6, and 7), with MEG concordant to resection margins for four of six cases (cases 1, 2, 3, and 7) of which three of four were seizure free (cases 2, 3, and 7). No cases had concordant MEG and interictal source coherence. Cases with MEG that had interictal HFO ESI and interictal HFO source coherence available were concordant (cases 2 and 7 included).

### FDG-PET

FDG-PET scans were conducted in 10 of 12 cases (cases 8 and 9 excluded). Focal areas of hypometabolism were reported in all 10 cases (cases 1, 2, 3, 4, 5, 6, 7, 10, 11, and 12) with FDG-PET concordant to the resection margins in 10 cases, of which six of 10 (cases 2, 3, 4, 7, 11, and 12 included; cases 1, 5, 6, and 10 excluded) were seizure free. Ictal source coherence maps were concordant with FDG-PET in all six cases with both data available (cases 2, 4, 5, 7, 10, and 12 included), with all cases but two achieving seizure freedom (case 5 and 10 excluded). FDG-PET was concordant with interictal source coherence for the case with both information available (case 4), with the patient also being seizure-free postop. FDG-PET was also concordant for two of three cases with interictal HFO ESI and interictal HFO source coherence available (cases 2 and 7 included), who became seizure-free post-surgery.

### Intracranial monitoring

This was performed in seven of twelve cases (cases 1, 2, 4, 5, 6, 7, and 10 included, whereas cases 3, 8, 9, 11, and 12 were excluded) with subdural grids and, or depth electrodes in five (cases 1, 2, 4, 5, and 7) and stereotactic EEG in two cases (cases 6 and 10).

### Pathology

Of the twelve cases with focal resections, four had focal cortical dysplasia (FCD) (case 2—FCDIIB, case 7—FCDIIA, case 8—FCD IIIB, and case 10—FCDIIB), four had neuronal tumors (cases 4, 5, 11: ganglioglioma; case 9, dysembryoplastic neuroepithelial tumor (DNET)). The tissue pathology for case 1 was described as having heterotopic grey matter in subcortical white matter; case 6 had subpial gliosis and oligodendrogliosis; and case 12 had gliosis and hippocampal sclerosis. The pathology for case 3 was unknown. All cases with recordable networks present on interictal HFO source coherence ESI had focal cortical dysplasia, with case 2 being FCD IIB, case 7 being FCD IIA, and case 8 being FCD IIIB. This may reflect the lesions for these cases being more superficial, allowing scalp EEG to successfully detect ripples. Otherwise, no correlation was found between the histopathology and the ictal source coherence ESI, interictal source coherence, interictal HFO ESI, or interictal HFO source coherence ESI.

### Postsurgery neurocognitive evaluation

Neurocognitive outcomes were available in 11 of the 12 cases. The postsurgical neurocognitive summaries together with the surgical procedure, pathology, and ILAE seizure outcome class for each case are depicted in Table 2.

**Table 2:** Post surgery neurocognitive evaluations.

Case (*n* = 12)	Age (Median 11; IQR 8.5)	Sex (7 female)	Surgical operation	Pathology	ILAE seizure outcome class at 3 years	Post surgery neurocognitive evaluation
1	17	M	R temporal lobectomy	Heterotopic gray matter	3	No post-surgical neuropsychological data
2	12	F	Right postcentral gyrus lesionectomy	FCD II B	1	Overall intact (Average) intelligence. Declines in visual–spatial skills (remained above average), auditory and visual memory, non-dominant (left) hand motor skills. Improved executive functioning on direct testing. Ongoing issues with anxiety and depression.
3	1	F	R posterior quadrant disconnection R temporal lobectomy	Unknown	1	Overall impaired (Extremely Low) intelligence (Intellectual Disability) with relative strength in receptive language and verbal memory (Average) and deficits in non-verbal skills. Query autism.
4	16	F	R occipital, parietal lesionectomy	Ganglioglioma	1	Overall, below average (Very Low) intelligence. Stable profile compared to pre-surgical with intact verbal reasoning and speed of information processing (both Low Average range) and weaknesses in nonverbal (visual–spatial) abilities, short-term memory, and left-sided fine-motor dexterity.
5	4	M	L cingulate lesionectomy	Ganglioglioma	4	Initial post-surgical assessment documented intact and age-appropriate (Average) verbal abilities but deficits in nonverbal skills, including visual–spatial abilities, nonverbal reasoning, visual processing speed, and visual motor integration. Problems with behavioral regulation and attentional control (i.e., aggressive behaviors, difficulty withholding impulses, difficulty avoiding distractions, struggling to focus and sustain his attention). Diagnosed with a Neurodevelopmental Disorder associated with medically condition and ADHD – combined presentation. Re-assessment after 5 years documented that verbal comprehension declined to Extremely Low, overall intellectual functioning was impaired (Extremely Low), as was adaptive functioning (daily life skills), and now instead of broad neurodevelopmental disorder met criteria for Intellectual Disability of moderate severity.
6	17	F	R anterior temporal lobectomy, R orbital frontal resection	Subpial gliosis, oligodendrogliosis	4	Overall stable, intact and age appropriate (Average) intelligence and cognition.
7	13	F	L post central gyrus lesionectomy	FCD II A	1	Stable nonverbal abilities (visual memory, processing speed, and right-hand dexterity) and improvement in visual-motor integration but decline (little development) in verbal abilities, working memory, auditory memory, and left-hand dexterity. Mild concerns about mood and withdrawal.
8	12	M	L gyrus rectus lesionectomy	FCD IIIB	1	Improved with Average intelligence and cognitive abilities; reflective of starting stimulants for ADHD + seizure freedom. Ongoing issues with executive functioning and blunted affect and lack of motivation.
9	10	F	R cingulate gyrus lesionectomy	DNET	1	Improved overall intelligence with improvement in visual skills (above average), processing speed, working memory, attention, and executive functioning. Stable profile otherwise with ongoing deficits in fine motor skills and visual memory.
10	8	M	R parietal lesionectomy	FCD IIB	4	1-year post-surgery: overall intelligence declined to Extremely Low (verbal comprehension, visual-constructional abilities, non-verbal reasoning, and verbal memory all declined). Increased impulsivity, distractibility, and difficulty sustaining attention. Diagnosed with Neurodevelopmental disorder associated with a medical condition, ADHD - inattentive presentation, and Developmental Coordination Disorder. Re-assessment 2 years later found greater than expected gains in non-verbal abilities (visual–spatial skills, fluid reasoning, visual working memory, and visual memory all Average) such that non-verbal intelligence now squarely Average. Continues to have deficits in slowed visual-motor processing speed, weak visual-motor integration, weak graphomotor skills, and verbal skills (expressive vocabulary, word finding difficulties, verbal fluency, higher-order verbal abstract reasoning). The following diagnoses were applicable: Attention-Deficit/Hyperactivity Disorder (ADHD), predominantly inattentive presentation, Developmental Coordination Disorder, Language Impairment in expressive language, and Learning Disability.
11	3	F	L temporal lobectomy	Ganglioglioma	1	Made developmental gains but relative standing compared to same-aged peers continued to be impaired. Along with deficits in adaptive functioning, met criteria for diagnosis of a Global Developmental Delay.
12	8	M	R temporal lobectomy	Gliosis, hippocampal sclerosis	1	One-year post-surgery: fluid reasoning, fine motor dexterity, visuomotor coordination and integration, and verbal memory declined while visual memory improved. Marked executive dysfunction and behavior dysregulation but improved from pre-surgical levels (now on ADHD medication). Diagnosis of Attention-Deficit/Hyperactivity Disorder-combined presentation continued to be relevant. Three years later, improvement in language (now Average) but ongoing deficits in attention, memory, with declines in reasoning skills and visual abilities (visual construction, visual motor integration, and visual motor processing speed). Diagnosed with Intellectual Disability of Mild Severity.

M, male; F, female; R, right; L, left; FCD, focal cortical dysplasia; DNET, dysembryoplastic neuroepithelial tumour.

## Discussion

The concept of seizure localization for presurgical evaluation being described in terms of overlapping zones (symptomatogenic, irritative, ictal onset, and EZ) should be held in tension by the observation that epilepsy is primarily a disorder of neural networks, where seizures may be entrained from any given part of the network and that interruption or modification of the network in any part will alter the seizure occurrence or expression (4, 50).

This study is novel because it describes how the combination of non-invasive, multimodal imaging techniques of the ictal onset, irritative and functional deficit zones, and ictal and interictal source connectivity maps of epileptic networks can be used together, to better understand the mechanisms of seizures in children undergoing epilepsy surgery.

Literature on ESI thus far has largely focused on estimating the ictal onset using only one source of maximum power. Epilepsy is a network disease, meaning that utilizing functional connectivity analysis with ESI can assist in more accurately estimating the ictal onset zone. However, a limited number of studies in the literature utilize functional connectivity analysis with ESI to estimate seizure onset zones (52, 54, 56–59, 63). In addition, the existing literature on utilizing functional connectivity analysis with ESI has primarily been in adults (54, 56, 58, 59) using LD-EEG or HD-EEG electrodes. The utility of LD-ESI with source coherence maps to localize the ictal onset zone in the surgical workup of medically refractory epilepsy in children has only been reported by a few centers (52, 57, 63). Functional connectivity analysis can estimate the causal relationships and direction of flow between brain regions to identify the driving hub of ictal networks. Functional connectivity in addition to ESI has been shown to significantly increase non-invasive localization of the ictal onset zone compared to ESI alone (56, 57). Currently, intracranial monitoring is the gold standard in determining the EZ for resection; however, intracranial monitoring is invasive and limited in its spatial sampling, with electrode placement being crucial to avoid sampling error. A future application for LD-ESI would be in guiding electrode placement for intracranial monitoring, to more accurately identify the epileptogenic zone for resection, specifically as there is a trend toward more use of minimally invasive stereo EEG depth electrodes instead of craniectomy with broader surface coverage using grids and strips.

### Ictal ESI source coherence

Six of seven source coherence maps included the surgical resection. Five of seven cases were seizure free postresection excluding case 5 (Class 4 outcome at 1 year) and case 10 (Class 4 outcome at 3 years). Cases were concordant with MRI, PET, ictal ESI (except case 10), interictal ESI, interictal source coherence ESI, interictal HFO ESI, and interictal HFO source coherence ESI where available. All cases had ictal ESI source coherence maps concordant with MEG, except case 5, in which MEG was discordant with all other data and the surgical resection. These findings support the hypothesis that interruption or modification of the epileptic network, at any part within the network, will alter seizure occurrence and expression (50).

A reasonable explanation for case 5 not achieving seizure freedom could be provided by the ictal source coherence map, as within it, the ictal onset zone network demonstrated a network involving the left central, temporal, and parietal regions, which extended beyond the surgical resection margins for the surgery done, a left cingulate lesionectomy, suggesting that while the EZ was included in the ictal onset zone network, it likely extended beyond the resection. In addition, while the source coherence map was concordant with MRI, PET, ictal ESI, and interictal ESI, it was discordant with the MEG, which localized to the contralateral perisylvian region. For case 10, the ictal source coherence map demonstrated an epileptic network involving the right occipital parietal temporal regions, which extended beyond the margins of the right parietal lesionectomy, again suggesting the EZ extended beyond the resection margin. The source coherence map was concordant with the MRI and PET and discordant with the ictal ESI which localized to the right temporal region. Our results provide support for LD-ESI using source coherence maps as both a practical and complementary method of localizing the ictal onset zone in the epilepsy surgical workup of children, when the results are concordant with MRI, interictal MEG, PET, ictal ESI, and interictal ESI.

### Interictal ESI source coherence

Interictal source coherence maps were generated for three cases (3, 4, and 8 included); however, cases 3 and 8 displayed generalized activity, which did not allow for localization of an ictal onset zone network. The interictal source coherence map for case 4 demonstrated a network involving the parietal occipital region, more evident in the right hemisphere. The interictal source coherence map for case 4 was concordant with MRI, PET, ictal ESI, and interictal ESI. This network included the surgical resection, and the patient was seizure-free post-operatively. While the interictal source coherence map for case 4 is promising, given that only one case within the study generated a source coherence map that allowed for localization, further research assessing the utility of interictal source coherence maps in the irritative zone localization is necessary. The estimation of interictal functional connectivity using non-invasive HD EEG may be a promising tool in the presurgical evaluation (60).

### Spike ripple network localization

Additionally, interictal HFOs have been presented as promising biomarkers of epileptogenic tissue, with most studies analyzing HFOs using HD-EEG and ESI, providing support for its use in epilepsy surgery workup (68–72). Only two studies thus far have utilized ESI with connectivity analysis to look at HFOs to estimate seizure onset zones, and both utilized LD-EEG and focused on specific patient populations, namely infantile spasms and sudden unexpected death in epilepsy (SUDEP) risk, respectively (75, 76). Our study is the first to assess the utility of LD-ESI to localize spike ripple networks using source coherence maps in the epilepsy surgery workup of children.

### Interictal HFO ESI

The results from our study align with previous studies assessing HFOs using ESI. Tamila et al. used HD-EEG and MEG to demonstrate that the ictal onset zone could be localized using HFOs through concordance with the surgical resection or intracranial recordings of 16 medically intractable focal epilepsy patients (72). Another study utilized HD-EEG to localize the ictal onset zone using HFOs in two children undergoing epilepsy surgery and found the localization concordant with intracranial EEG (71).

Our research demonstrated similar results to previous studies in literature, by utilizing LD-EEG to conduct interictal HFO ESI in three patients (2, 7, 8, included). No distinct HFO band was seen on STFFT for cases 1, 6, 9, and 11. This was likely due to the inability of scalp EEG to detect HFO from deep sources, as in these cases the EZ originated from the temporal lobe or cingulate gyrus (77). Interictal HFO ESI was located within the surgical resection margins of two of three (cases 2 and 7) and concordant with MRI, FDG-PET, MEG, ictal ESI, ictal source coherence, and interictal ESI. However, interictal HFO ESI for case 8 localized to the left temporal region, which was not concordant with ictal ESI, which localized to the left frontal region, outside of the resection margin. All three cases were seizure-free at the 1-year mark postoperatively.

### Interictal HFO source coherence ESI

Literature on ESI connectivity using HFOs to localize seizure onset zones is scarce, with the two studies focusing on specific patient populations. Samfira et al. found fast ripple global efficiency increased during spasms in 14 patients with infantile spasms (75). Tufa et al. looked at HFO EEG coherence networks in 8 SUDEP and 14 non-SUDEP epileptic patients and demonstrated support for this method as a non-invasive biomarker of SUDEP risk (76). Our study is the first to look at the utility of interictal HFO source coherence maps constructed using LD-ESI in the epilepsy surgical workup of children. For the three patients with interictal HFO ESI with distinct HFO bands visualized on SFFT, interictal HFO source coherence maps were created (2, 7, and 8 included). For two cases (2, 7, included) both were concordant with MRI, PET, MEG, ictal ESI, ictal source coherence, interictal ESI, and interictal HFO ESI. Two of three cases included surgical resection and were seizure free 1-year postoperatively. This study supports the utility of LD-ESI analysis of HFOs using source coherence maps as both a practical and complementary method of assessing the ictal onset zone localization in the epilepsy surgical workup of children, when the results are concordant with MRI, interictal MEG, PET, ictal ESI, and interictal ESI.

### Limitations

Our study is limited by its use of retrospective data from a small cohort of patients.

We recognize the lower spatial resolution of LD ESI may miss anatomical details in source reconstruction. Another limitation is that while 25 channels were applied in 11/12 cases either additional midline or subtemporal electrodes were used. A uniform use of electrodes may have improved the inverse problem solution. Not all diagnostic modalities were available for each patient. Our cohort also lacked MRI-negative subjects, which can present as more challenging in assessing surgical candidacy. The cohort was heterogeneous in etiology by histopathology, which may have influenced how epileptic networks were generated. Histopathology is an important determinant of seizure freedom following epilepsy surgery, meaning the heterogeneity of histopathology included may have impacted the surgical outcomes in our cohort. Pathologies associated with the worst outcomes include FCD I, mild MCD other, glial scar, and no histopathological lesion (7). Pathologies with poorer outcomes included in this study were MCD other, glial scar, and no histopathological lesion.

## Conclusion

ESI with connectivity analysis to estimate the ictal onset zone and spike ripple network presents itself as a potentially promising non-invasive diagnostic technique within the epilepsy surgical workup of the pediatric population, complementing existing diagnostic tools. A future application for LD-ESI would be in guiding electrode placement for intracranial monitoring. Despite a current growing body of research on ESI and HFOs, there is a lack of studies combining connectivity analysis with ESI, which can provide important further information given the nature of epilepsy as a network disease. Our results of the study provide proof of principle supporting the utility of LD-ESI of the ictal onset zone and spike ripple network using source coherence maps as a practical method complementary to ictal ESI in the epilepsy surgical workup of the pediatric populations. Given the novelty of this technique and the present promising support for it, there is a need for further research on LD-ESI of the ictal onset zone and spike ripple network using source coherence maps utilizing larger cohorts of patients to allow for the use of inferential statistics to validate the descriptive findings we present.

Further studies utilizing a more homogenous cohort, such as in distinct populations and patient subsets, are also needed. In addition, further directions of research include the utility of source coherence maps in guiding electrode placement for intracranial monitoring to more accurately identify the EZ for resection.

## Data Availability

The original contributions presented in the study are included in the article/supplementary material, further inquiries can be directed to the corresponding author.
